# Supplementary material for: Computational Model Exploring Characteristic Pattern Regulation in Periventricular Vessels
Source: Life (Basel). 2022 Dec 9;12(12):2069. doi: 10.3390/life12122069 (PMC9788473; doi:10.3390/life12122069)
Supplement: Supplementary file 1 [file life-12-02069-s001.zip › life-1981454-supplementary.pdf]

Supplementary Material

Computational Model Exploring the Regulation for Characteristic Patterns in Periventricular Vessels

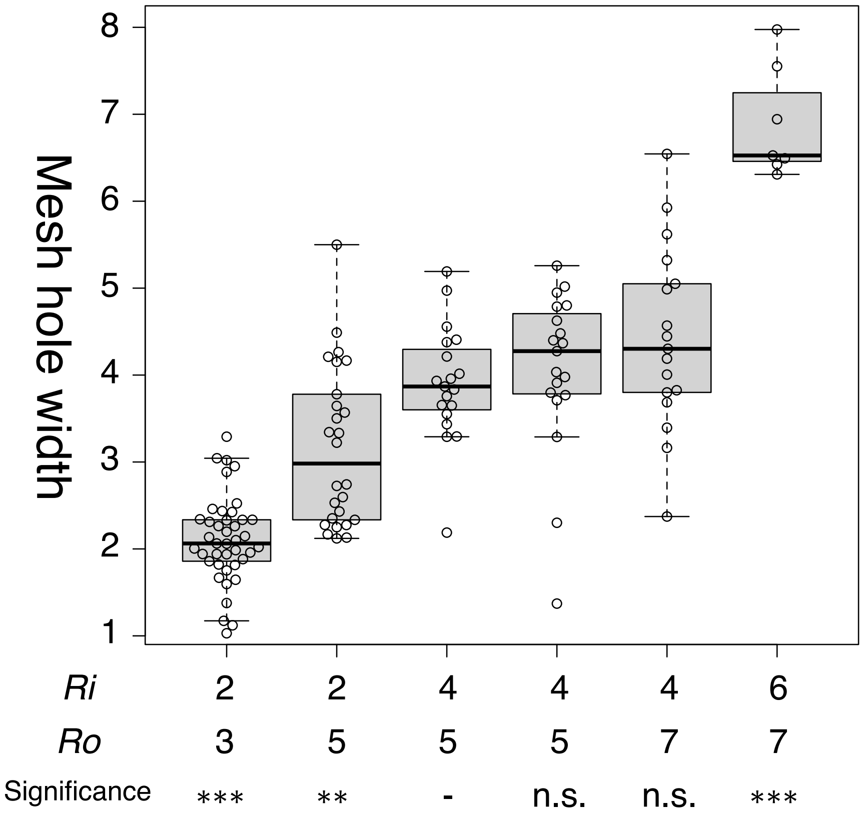

Figure S1. Quantification of inter-vessel spacing depending on repulsive range in Figure 4. Dots indicate mesh hole width in Figure 4f (*Ri* = 2, *Ro* = 3), 4g (*Ri* = 2, *Ro* = 5), 4b (*Ri* = 4, *Ro* = 5), 4d (*Ri* = 4, *Ro* = 5 with random initial cell arrangement), 4h (*Ri* = 4, *Ro* = 7), and 4i (*Ri* = 6, *Ro* = 7). The significant differences in mesh hole widths from those in Figure 4b are indicated as *** $p < 0.001$ and ** $p < 0.01$. n.s.: Not significant.

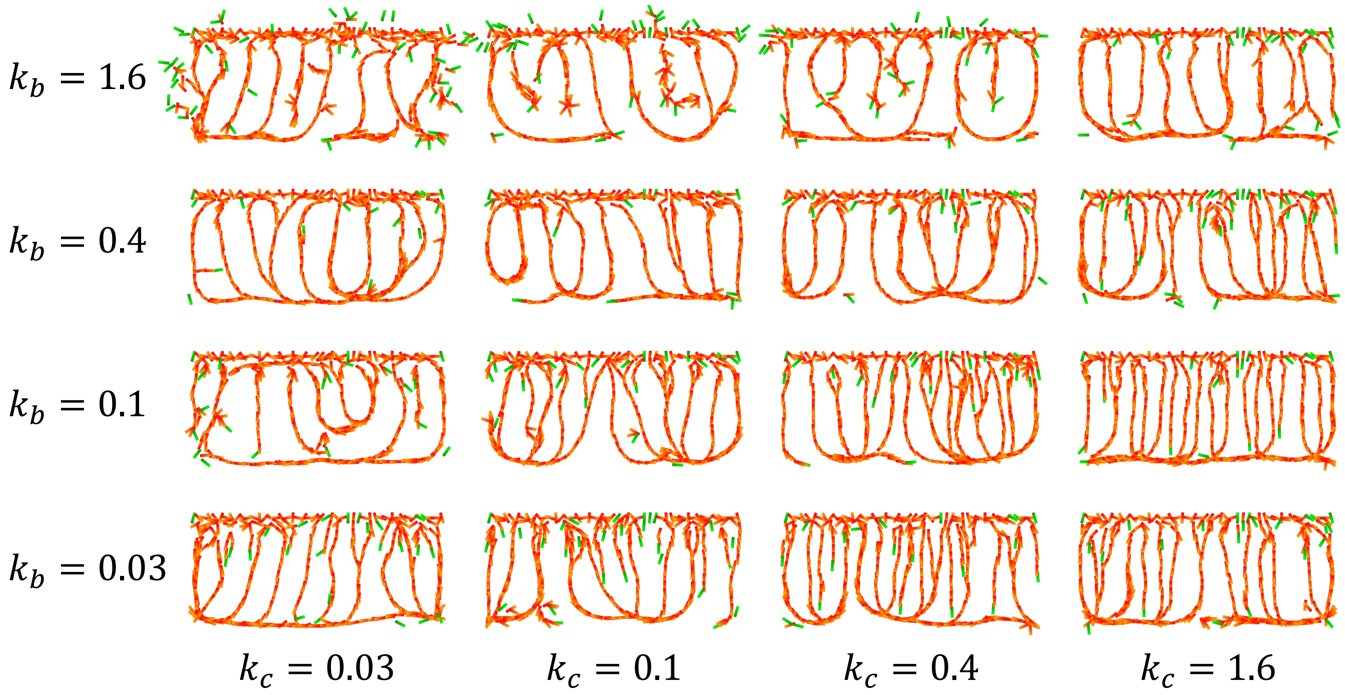

Figure S2. Parameter dependency of patterns in CP vessel simulation. The conditions are as in Figure 3f except for k_c and k_b. Calculation results at $t = 150$ are shown. If directed migration dominates over repulsion, sprouting from the pial vessel is excessively promoted, resulted in an excess number of vertical branches. The balance between directed migration and repulsion generates almost equally spaced vessels. The intense repulsion inhibits anastomosis and does not promote branch formation but disrupts the CP pattern.

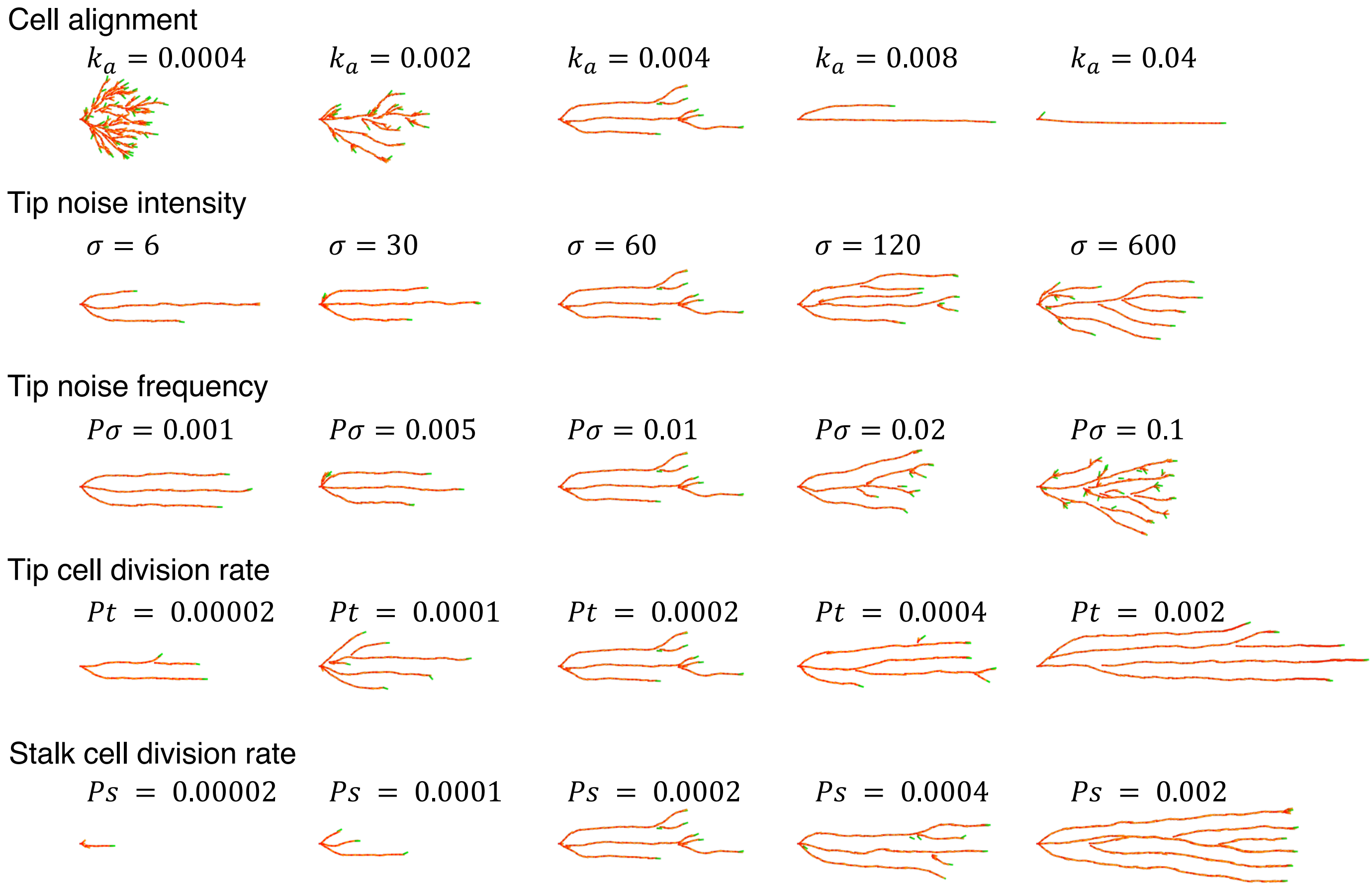

Figure S3. Effect of each parameter on branch formation. The parameter values indicated in each image were changed from the parameter set used in Figure 3f. The initial cell was placed with the migratory direction to the right. The forced 1direction was set **c** = (1,0). Calculation results at t = 240 are shown.
